# Cognitive management in a digital world

**DOI:** 10.18632/aging.203324

**Published:** 2021-07-13

**Authors:** Michelle Gray, Joshua L. Gills, Jordan M. Glenn

**Affiliations:** 1 University of Arkansas, Department of Health, Human Performance, and Recreation, Fayetteville, AR 72701, USA; 2 Neurotrack Technologies, Inc., Redwood City, CA 94063, USA

**Keywords:** Alzheimer's disease, dementia, cognitive decline, digital intervention

Alzheimer’s disease (AD) and related dementias (ADRD) affect an estimated 6.2 million older Americans [1]. AD is the 5^th^ leading cause of death [1] and is also the leading cause of disability among US older adults. Currently, no cure exists for ADRD and pharmacological treatments are largely ineffective when diagnosed correctly. Approximately 76% of patients with cognitive decline are not appropriately diagnosed [2]. The reasons individuals go undiagnosed are multifactorial; however, they are partly attributed to the inability of primary care physicians to recognize symptoms and failure to complete appropriate screening tools until late in the disease’s manifestation [2]. Currently, in the US, performing routine screening assessments for cognitive function or decline is not recommended due to the lack of effective treatments [2]. These common practices result in delayed identification and treatment for cognitive decline. Early detection and treatment through interventions could add nearly 5 ADRD-free years to a person’s life [3] and reduce associated health care costs by 44% [1]. Nonetheless, interventions are futile if not begun in the early stages of the disease progression. Thus, early detection is warranted to adequately slow cognitive decline. The concept of early detection is simple, but the reality is often complex based on limited access to care. Additionally, many standard neuropsychological assessments are not sensitive enough to detect early cognitive changes. In the US, 20 states have been identified as “dementia neurology deserts,” indicating a significant gap between the patient needs and available treatment options [4]. Furthermore, the current global pandemic has thwarted the effort to combat early cognitive decline even more.

Due to COVID-19, traditional healthcare practices were forced to rethink the use of telehealth. Telehealth programs not only increased their usage among primary care, but also among neuropsychological providers. This transition also increased the demand for valid and reliable digital cognitive testing has increased significantly. To date, there are a limited number of validated digital cognitive assessments and of these, most are not readily available to the public. Our group has recently validated a short (5-minute) digital cognitive measurement assessing declarative memory [5–7]. This assessment is positively correlated with well-established cognitive screening tools and reliably predicts current cognitive status [6,7]. Remote cognitive screenings provide health care professionals with an additional tool when assessing cognitive decline, are time efficient, and allow for physicians to give information on various treatments to combat further abnormal decline.

Interventions often use a single-domain (e.g., pharmacological, exercise, etc.) approach to improve cognitive function. Results from these interventions were ineffective after observing changes in physical activity, nutrition, cardiovascular health, or cognitive training on improving cognitive outcomes [8]. These results are not surprising considering the cause of ADRD is multifactorial. Thus, more recently longitudinal multi-domain interventions have resulted in positive changes in cognitive outcomes and risk of cognitive decline. It should be noted, however, that many of the primary outcome changes have been non-significant. One common thread of these interventions is the age of participants. Many have targeted older adults (>70 years) with existing underlying cognitive impairment and/or decline. At this point in a person’s life, it may be too late for effective interventions to significantly impact cognitive outcomes. Therefore, interventions should target improving positive risk factors associated with cognitive decline rather than cognition as a singular outcome among high-risk individuals.

Our group has recently begun a digital multi-domain intervention with the goal of reducing cognitive impairment risk of 45-75 year-old cognitively normal adults (NIH SBIR R44AG063672). We were intentional in our selection of research participants to examine changes in risk factors over a 2-year intervention. The project includes a digital multi-domain intervention with a personalized health coach providing direct counselling on physical activity practices, dietary habits, social engagement, and cognitive training. A personalized health coach model was chosen because not all individuals have the same needs. Accordingly, a multi-domain intervention providing individualized counselling is ideal for improving overall health outcomes and improving risk factors for future cognitive decline [8]. Additionally, focusing on lifestyle changes in mid-life and relating those changes to the improvements in cognitive performance in late life is an important and missing piece of the puzzle.

Behavior modification programs are difficult for individuals to adopt due to a myriad of reasons including insufficient time, perceived cost, and knowledge of specific changes. Virtual health coaching provides the knowledge to change various aspects of health, including focusing on healthy eating, physical activity participation, and social engagement. Doing so in a virtual environment is more cost effective than paying for a gym membership or personal training. In theory, this type of intervention is both efficacious and cost effective; however, has not been shown to significantly improve overall health outcomes or delay cognitive decline. Thus, further research is warranted, but results are promising.
